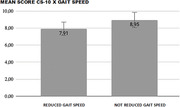# Do patients with early‐stage cognitive impairment have an increased risk to have a reduction in walking speed? A cross‐sectional study

**DOI:** 10.1002/alz.089259

**Published:** 2025-01-09

**Authors:** Pedro de Castro Lopes, Amanda Aparecida Oliveira Leopoldino, João Carlos Barbosa Machado, Maira Tonidandel Barbosa, Luana Rodrigues Garcia, Bianca Pessoa Aguiar, Júlia Caroline Barbosa de Souza, João Pedro Neres Antunes Ferreira

**Affiliations:** ^1^ Rede Mater Dei de Saúde, Belo Horizonte, Minas Gerais Brazil; ^2^ Faculdade Ciências Médicas de Minas Gerais, Belo Horizonte, Minas Gerais Brazil

## Abstract

**Background:**

Gait speed is an important measure in the evaluation of elderly patients. The gait speed reduction is associated with high‐impact outcomes such as: loss of functionality, frailty and increased mortality. Several clinical conditions lead to a reduction in gait speed. In patients with advanced dementia, it could be associated with disability. However, the relationship between cognition and motor function is not well known, especially in the early stages of dementia and further studies are needed. It is also important to understand whether patients with mild cognitive impairment have increased risk of a worse gait speed.

**Method:**

This is a cross‐sectional study in which 87 community‐dwelling elderly people were included and evaluated in relation to the presence of cognitive impairment and gait speed. Patients with severe cognitive impairment, assessed by a score less than 6 in the CS‐10 (Point Cognitive Screening) were excluded. Individuals with a score between 6 and 7 on the CS‐10 were considered to have mild or moderate cognitive impairment and participants with a score greater than 7 on this test were considered to have no cognitive impairment. In order to evaluate gait speed, individuals were stimulated to walk for 4 meters, and those with a walking speed lower than 0.82 m/s were considered to have it reduced. With the aim of estimate the association between the characteristics, Pearson’s chi‐square test was used. If the p‐value was lower than the significance level of 0.05, it is possible to conclude the association between the lower walking speed and a worse score in CS‐10.

**Result:**

Statistical analysis showed an association between the reduction in gait speed and CS‐10 score (p‐value 0.041). Patients who had lower score on the cognitive assessment are likely to have a lower gait speed. The mean CS‐10 score for patients with gait impairment is 7.91, while for patients without gait impairment is 8.95 points. The Kruskal‐Wallis test indicates that there is a significant difference in the results (p‐value 0.016).

**Conclusion:**

Individuals with mild cognitive impairment have an increased risk of developing reduced gait speed.